# Efficacy of Probiotics Versus Tetracycline Fibers as Adjuvants to Scaling and Root Planing on Interleukin 1β Levels in Type 2 Diabetic Patients With Periodontitis: A Clinical and Biochemical Study

**DOI:** 10.7759/cureus.50968

**Published:** 2023-12-22

**Authors:** Pranavi Gullapelli, Rekha R Koduganti

**Affiliations:** 1 Department of Periodontics, Panineeya Mahavidyalaya Institute of Dental Sciences & Research Centre, Hyderabad, IND

**Keywords:** diabetes, il1β, placebo, probiotics, tetracycline fibers, elisa, gcf, scaling and root planing, clinical parameters, periodontitis

## Abstract

Background

Periodontitis, a chronic inflammatory disease, is triggered by the plaque biofilm culminating in periodontal attachment loss, bone loss, and tooth loss. Diabetes, a globally prevalent disease, causes an increased inflammatory response to the microflora associated with periodontitis. It has been observed that the link between these two diseases is bidirectional. Tissue repair is impaired in diabetic patients with periodontitis. Local drug delivery systems selectively target the inflamed sites contrary to systemic antibiotics which lead to resistance and many other adverse effects. Probiotics aid in the growth of beneficial microorganisms and have immunomodulatory effects on the host. Tetracyclines have anti-collagenase properties and reduce the bacterial load, curbing the progression of periodontitis. Interleukin (IL) 1β, a strong marker of periodontal tissue destruction, plays a pivotal role in inflammation, immune regulation, and bone resorption in periodontitis. This study evaluated and compared the benefits of probiotics and tetracycline fibers when used as adjunctive tools after scaling and root planing (SRP) on IL1β levels in type 2 diabetic patients with periodontitis.

Methodology

A total of 36 patients participated in this study. Group I included 12 patients with periodontitis and uncontrolled diabetes (HbA1c levels >7). After SRP, six patients received tetracycline fibers (IA), and six patients received probiotics (1B) as locally delivered agents. Group II included 12 patients with periodontitis and diabetes under control (HbA1c levels 6-7%). After SRP, six patients received tetracycline fibers (IIA), and six patients received probiotics (IIB) as locally delivered drugs (test groups). Group III, the control group, included 12 patients with periodontitis only, wherein a placebo was used as a local drug delivery (LDD) after SRP. The clinical parameters, such as plaque index, gingival index, and probing pocket depth, were recorded preoperatively and at eight and 12 weeks after non-surgical periodontal therapy. IL1β levels were assessed by enzyme-linked immunosorbent assay at baseline and six weeks after SRP.

Results

On intra and intergroup comparison, all groups showed improvement in both the clinical and biochemical parameters but significant results were seen in Group IIA (p < 0.01) when compared to the other groups.

Conclusions

Group II (well-controlled diabetics) performed significantly better than the other groups, which was followed by Group III. The use of LDDs as adjunctive tools after SRP was not beneficial in Group I (uncontrolled diabetics).

## Introduction

Plaque is a harbinger of periodontitis. Mature plaque aids in the progression of the disease resulting in excessive attachment and bone loss [1]. Diabetes mellitus (DM) may considerably alter the prevalence, severity, and response of periodontal disease to periodontal treatment. Therefore, DM was included in the clinical classification of periodontitis as a descriptor in the staging and grading process [2]. Scaling and root planing (SRP) is the initial treatment protocol. Initially, systemic antibiotics were used as adjuncts, but due to their shortcomings such as resistance and other side effects, they were replaced by local drug delivery (LDD) systems [3].

Tetracyclines have gained popularity due to their anti-collagenase and bacteriostatic effects, and probiotics due to their immunomodulatory and antioxidant properties. Hence, tetracyclines and probiotics have been researched of late. Interleukin (IL) 1β, a pro-inflammatory marker, is frequently detected in the saliva and gingival crevicular fluid (GCF) of patients with periodontitis compared with healthy controls. Hence, this study compared the benefits of using tetracyclines and probiotics as LDDs after SRP on clinical parameters and IL1β levels in diabetic patients with periodontitis.

## Materials and methods

**Figure 1:**
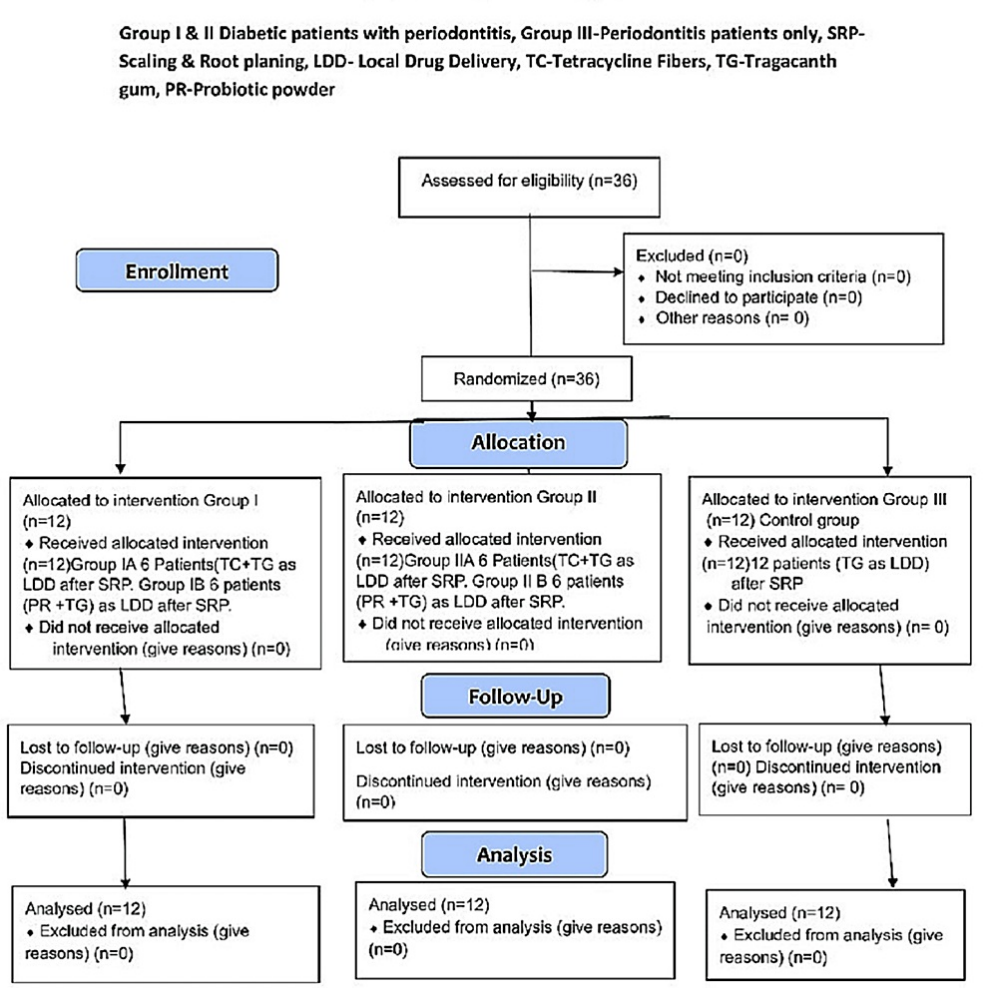
CONSORT flow diagram.

This study was designed as a randomized, prospective, parallel-arm, interventional clinical trial. It was conducted according to the guidelines and after obtaining approval from the institutional ethical committee of Panineeya Mahavidyalaya Institute of Dental Sciences and Research Centre (PMVIDS&RC IEC PERIO/DN332-20). Written informed consent was obtained from all patients after a thorough explanation of the nature, benefits, and risks of the clinical investigation and associated procedures. The trial was also registered (CTRI/2021/05/033474) (Figure 1).

**Figure 2:**
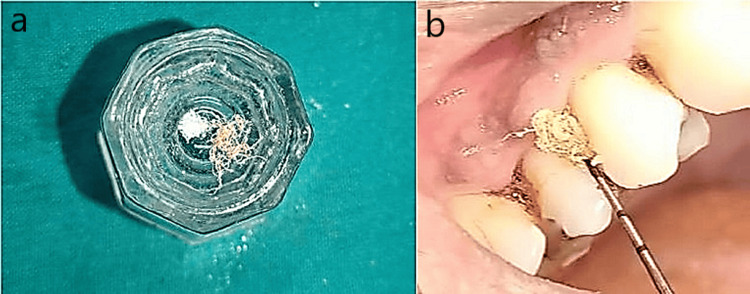
Group Ia and Group IIa. a: Tetracycline fibers and tragacanth gum in a Dappen dish. b: Insertion of tetracycline fibers + tragacanth gum into the pocket.

**Figure 3:**
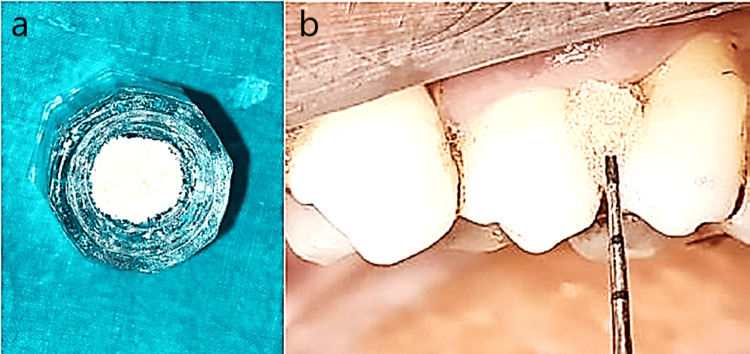
Group Ib and Group IIb. a: Probiotic powder and tragacanth gum in a Dappen dish. b: Insertion of probiotic + tragacanth gum into the pocket.

A total of 24 patients with periodontitis and diabetes and 12 patients with periodontitis only were selected. Group I included 12 patients with type 2 uncontrolled diabetes (HbA1c levels >7), who underwent non-surgical periodontal therapy, followed by administration of tetracycline fibers (Periodontal Plus AB - Advanced Biotech Products, Tamilnadu, India) mixed with 2 mg of tragacanth gum (TC) powder or xanthan gum (which is resorbable and has been used as a carrier to increase the retentivity) and 0.2 mL of saline as LDD in six patients (IA) (Figures 2a, 2b). Probiotic powder mixed with 2 mg of TC powder plus 0.2 mL of saline was employed as a local drug in six patients (IB) (Figures 3a, 3b).

In Group II, 12 patients with well-controlled diabetes (HbA1c levels 6-7%) underwent the same treatment protocol as Group I, wherein after SRP, six patients were administered tetracycline fibers (IIA) and six patients were administered probiotics (IIB) (Figures 3a, 3b).

**Figure 4:**
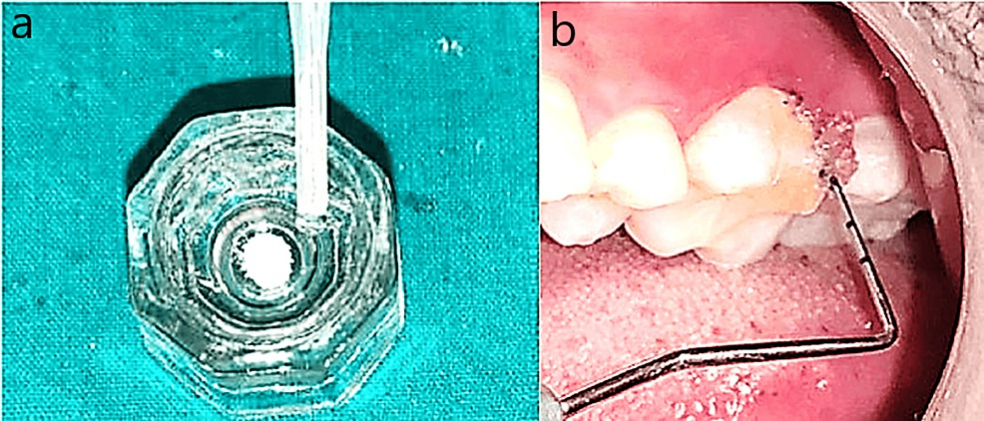
Group III (control group). a: Tragacanth gum + saline in a Dappen dish. b: Insertion of tragacanth gum + saline into the pocket.

Group III included the control group wherein 12 patients with periodontitis participated. After SRP, a placebo (2 mg of TC powder mixed with 0.2 mL of saline) was administered into the pocket (Figures 4a, 4b).

Inclusion and exclusion criteria

For diabetes, patients were segregated based on their HbA1c levels into uncontrolled (HbA1c >7%), allocated to Group I, and controlled (HbA1c 6-7%), allocated to Group II. For periodontitis, 20 teeth needed to be present with probing pocket depths (PPDs) of at least 5 mm in one or more sites. Pregnant and lactating women, smokers, patients with any autoimmune or systemic disorder other than type II diabetes, use of medicines or antibiotics three months prior, and patients with a history of periodontal treatment within six months were excluded from the study.

Method of local drug delivery administration

Patients were motivated about oral hygiene maintenance. A full‑mouth SRP procedure was performed. Locally, the drug was introduced into the pocket. Then, a periodontal dressing was applied for a week. Patients were asked not to disturb the treated sites for a week. At one week of recall, any adverse reactions to the drug were ruled out. The primary and secondary outcome measures were recorded at the stipulated time intervals.

**Figure 5:**
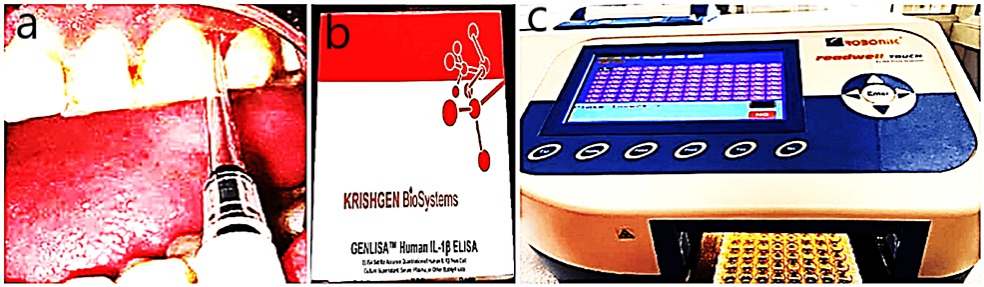
Estimation of IL1β. a: Collection of gingival crevicular fluid. b: Enzyme-linked immunosorbent assay reagent kit. c: Enzyme-linked immunosorbent assay reader.

Biochemical parameters included IL1β levels (primary outcome measure) which were assessed in the GCF by enzyme-linked immunosorbent assay (ELISA) at baseline and six weeks postoperatively. Overall, 2 µL of GCF was collected from the drug-delivered pocket using a calibrated pipette and was stored in 0.1 mL of phosphate buffer solution which was collected in an Eppendorf vial. These samples were stored at -20°C until the assay was performed (Figures 5a-5c).

Clinical parameters such as plaque index (PI), gingival index (GI), and PPD (secondary outcome measures) were recorded preoperatively and eight and 12 weeks after SRP using the Williams periodontal probe. An acrylic stent with marking was used at baseline and at eight and 12 weeks to measure the PPD for accurate and standardized measurements.

Statistical analysis

To determine a difference in IL1β levels between the groups at 80% power, 1% alpha error, and 90% confidence interval, 12 patients had to be included in each group. The analysis was done using SPSS Statistics version 23.0 (IBM Corp., Armonk, NY, USA). One-way analysis of variance (ANOVA) and Tukey’s test were performed for intergroup comparison. Repeated-measures ANOVA, Bonferroni test, and paired-sample t-test were performed for intragroup comparison. P-values <0.05 were considered statistically significant.

## Results

Intragroup and intergroup comparison

Within the groups, it was observed that the PI values were statistically significant only in Group IIA (p < 0.01). The PI values between the groups also showed highly significant improvement only in Group IIA, wherein the PI values decreased from baseline (0.6667) to 12 weeks (0.3333) (p < 0.01) (Table 1).

**Table 1:** Intragroup and intergroup mean comparison of the plaque index at different time intervals. The data are represented as mean ± standard deviation. Between-group comparisons were done by one-way analysis of variance (ANOVA) and Tukey’s test. Within-group comparisons were done by repeated-measures ANOVA and Bonferroni test. *: Significant at p < 0.05.

	Baseline		8 weeks		12 weeks		P-value
Intragroup	Mean	SD	Mean	SD	Mean	SD	
IA	1.3333	0.51640	1.5000	0.54772	1.1667	0.40825	0.36
IB	1.3333	0.51640	1.5000	0.54772	1.1667	0.40825	0.36
IIA	1.3333	0.51640	0.3333*	0.51640	0.3333*	0.51640	0.01*
IIB	1.1667	0.40825	1.0000	0.00000	1.0000	0.00000	0.36
III	1.2500	0.45227	1.3333	0.49237	1.1667	0.38925	0.33
	Baseline-8 weeks		Baseline-12 weeks		8 weeks-12 weeks		
Intergroup	Mean	SD	Mean	SD	Mean	SD	
IA	0.0000	0.00000	0.3333	0.51640	0.3333	0.51640	
IB	0.0000	0.00000	0.1667	0.40825	0.1667	0.40825	
IIA	0.6667	0.51640	1.0000	0.63246	0.3333	0.51640	
IIB	0.5000	0.54772	0.6667	0.51640	0.1667	0.40825	
III	0.1667	0.38925	0.3333	0.49237	0.1667	0.57735	
P-value	<0.011*		<0.051		<0.928		

The GI values within the groups were also significant only in Group IIA, and similar results were seen between the groups wherein the GI values decreased from baseline (0.8333) to 12 weeks (0.3333) (p < 0.01) (Table 2).

**Table 2:** Intragroup and intergroup mean comparison of the gingival index at different time intervals. The data are represented as mean ± standard deviation. Between-group comparisons were done by one-way analysis of variance (ANOVA) and Tukey’s test. Within-group comparisons were done by repeated-measures ANOVA and Bonferroni test. *: Significant at p < 0.05.

	Baseline		8 weeks		12 weeks		P-value
Intragroup	Mean	SD	Mean	SD	Mean	SD	
IA	1.6667	0.51640	1.6667	0.81650	1.6667	0.81650	1.00
IB	2.0000	0.63246	2.1667	0.40825	1.8333	0.75277	0.36
IIA	1.5000	0.54772	0.3333	0.51640	0.1667	0.40825	0.01*
IIB	1.5000	0.54772	1.5000	0.54772	1.1667	0.40825	0.07
III	1.5000	0.52223	1.4167	0.51493	1.4167	0.51493	0.58
	Baseline-8 weeks		Baseline-12 weeks		8 weeks-12 weeks		
Intergroup	Mean	SD	Mean	SD	Mean	SD	
IA	0.6667	0.51640	0.6667	0.51640	0.0000	0.00000	
IB	0.0000	0.00000	0.1667	0.40825	0.1667	0.40825	
IIA	0.8333	0.40825	1.1667	0.40825	0.3333	0.51640	
IIB	0.8333	0.40825	1.0000	0.63246	0.1667	0.40825	
III	0.1667	0.38925	0.5000	0.67420	0.3333	0.49237	
P-value	<0.00*		<0.029*		<0.559		

Regarding probing depth, statistically significant results were seen only in Group IIA within and between the groups. Baseline probing depth values (1.16) increased at eight weeks (2.66), following which they decreased at 12 weeks (1.00) which was significant (p < 0.01) (Table 3).

**Table 3:** Intragroup and intergroup mean comparison of probing depth at different time intervals. The data are represented as mean ± standard deviation. Between-group comparisons were done by one-way analysis of variance (ANOVA) and Tukey’s test. Within-group comparisons were done by repeated-measures ANOVA and Bonferroni test. *: Significant at p < 0.05.

	Baseline		8 weeks		12 weeks		P-value
Intragroup	Mean	SD	Mean	SD	Mean	SD	
IA	5.6667	0.51640	5.3333	0.51640	5.1667	0.75277	0.20
IB	6.3333	0.51640	6.1667	0.40825	6.0000	0.63246	0.36
IIA	5.8333	0.40825	4.6667	0.51640	3.6667	0.51640	0.000*
IIB	5.5000	0.54772	5.3333	0.51640	4.8333	0.75277	0.10
III	5.4167	0.51493	5.3333	0.49237	5.0000	0.60302	0.19
	Baseline-8 weeks		Baseline-12 weeks		8 weeks-12 weeks		
Intergroup	Mean	SD	Mean	SD	Mean	SD	
IA	0.3333	0.51640	0.5000	0.83666	0.1667	0.40825	
IB	0.1667	0.40825	0.3333	0.81650	0.1667	0.40825	
IIA	1.1667	0.75277	2.1667	0.40825	1.0000	0.89443	
IIB	0.1667	0.40825	0.6667	0.81650	0.5000	0.54772	
III	0.0833	0.28868	0.4167	0.51493	0.3333	0.49237	
P-value	<0.001*		<0.000*		<0.081		

**Figure 6:**
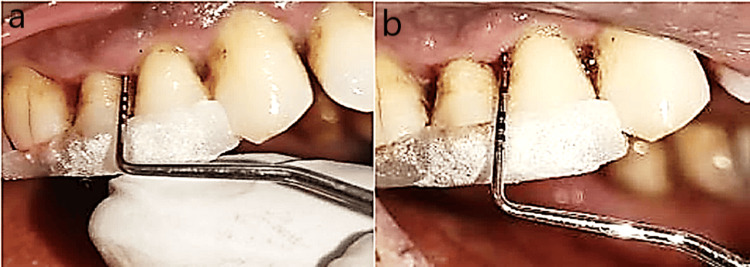
Estimation of probing pocket depth. a: Preoperative probing depth. b: Probing depth after eight weeks.

The same findings related to the PPD are depicted in Figure 6a and Figure 6b.

IL1β levels were also significant within the groups in Group IIA with mean values of 275.22 and 145.5 at baseline and six weeks, respectively (p < 0.01). Similar results were seen between the groups in Group IIA, wherein the difference from baseline to six weeks was 129.62 and was highly significant (p < 0.01) (Table 4).

**Table 4:** Intragroup and intergroup mean comparison of IL1β at different time intervals. The data are represented as mean ± standard deviation. Between-group comparisons were done by one-way analysis of variance (ANOVA) and Tukey’s test. Within-group comparisons were done by repeated-measures ANOVA and Bonferroni test. *: Significant at p < 0.05.

	Baseline		6 weeks		P-value		Baseline-6 weeks	
Intragroup	Mean	SD	Mean	SD		Intergroup	Mean	SD
IA	317.2417	40.69616	278.0433	10.76322	0.05	IA	22.5317	8.44932
IB	321.8333	35.65304	277.8917	16.21062	0.08	IB	21.6083	8.64353
IIA	275.2200	12.93533	145.5950	6.49962	0.000*	IIA	129.6267	14.60421
IIB	276.4383	13.55273	251.0900	47.24758	0.20	IIB	45.0150	5.04368
III	288.9750	14.66644	275.7017	22.94420	0.07	III	41.8408	4.00618
P-value	<0.000*		<0.000*			P-value	<0.00*	

## Discussion

Periodontitis and diabetes are chronic inflammatory diseases that are interlinked. Both diseases are globally prevalent. In diabetic patients, the risk of contracting periodontitis and vice versa is increased two to three times when compared to other patients due to the increased inflammatory burden which affects the glycemic levels and periodontal microflora [4]. Hyperglycemia stimulates the release of free radicals, which, in turn, cause cell death. Advanced glycation end products (AGEs), which are the hallmark of diabetes, cause excessive tissue injury due to their interaction with inflammatory mediators. Tumor necrosis factor-alpha antagonizes the action of insulin along with other cytokines such as IL6 and IL1 [5,6].

Non-surgical periodontal therapy has been the cornerstone of periodontal treatment, and it has been observed that SRP in diabetic patients with periodontitis leads to a reduction in glycosylated hemoglobin levels [7,8].

Many adjunctive modalities have been introduced to improve the treatment outcome after SRP in diabetic patients with periodontitis. Systemic antimicrobials were used earlier but have been avoided of late due to their adverse effects. In a meta-analysis, the effects of systemic antibiotics when used as adjuncts to non-surgical periodontal therapy (NSPT) were compared with NSPT alone in diabetic patients with periodontitis. Twelve studies met the inclusion criteria, nine of which provided data that allowed their inclusion in meta-analyses. The meta-analyses showed no significant effect of systemic antibiotics when used as adjuncts to NSPT on the reduction of HbA1c levels [9]. LDD systems have been employed after SRP as adjunctive tools with moderate success. The main advantage of LDD systems is the ease of application as well as very minimal side effects. This study assessed and compared tetracycline and probiotics (test groups) to the control group wherein only TC gum was placed as an LDD into the periodontal pocket. TC gum has been used in papermaking, cosmetics, and textile industries. It has also been used as a food additive and has been declared safe to use by the Food and Drug Administration. As TC gum is a polymer, the release of medicaments that use it as a carrier is slow, as they are encapsulated by the polymer, ensuring a constant release of the drug over time and following the zero-order kinetics [1].

A study by Boyeena et al. compared the efficacy of sub-gingivally delivered probiotics and tetracycline fibers individually and probiotics in combination with tetracycline fibers after SRP. TC was used as a carrier in all three groups. The clinical parameters were recorded before and 45 days after LDD administration. Microbiologic analysis was done by collecting plaque samples which were analyzed at the above-mentioned time intervals. They concluded that the colony-forming units were reduced in all three groups. Related to the clinical parameters the group that used probiotics in combination with tetracycline fibers performed better than the other two groups validating the benefits of using tetracycline and probiotics together [1].

Another study evaluated the clinical and metabolic effects of xanthan-based chlorhexidine (CHX) gel used as an adjunct to NSPT in type II diabetic patients with chronic periodontitis. The enrolled patients with glycated HbA1c levels >6% and moderate-to-severe periodontitis were equally split into two groups, namely, test and control. The test group was treated with NSPT in the control and NSPT + xanthan-based chlorhexidine gel in the test group. It was observed that both the fasting blood sugar and HbA1c levels markedly decreased in the test group after six months, supporting the role of xanthan-based CHX gel after NSPT [10].

In this study, TC gum was used in all three groups as a carrier but was used as a monotherapy in the control group (Group III). The control group showed better results for PI, GI, and PPD when compared to Group I (uncontrolled diabetics).

Tetracyclines are noted for their anti-inflammatory and anti-collagenase activities and have also been shown to promote the attachment of fibroblasts to the root surface [11]. Pharmacokinetic studies of Periodontal Plus AB revealed that the release rate is in a multimodal manner with zero-order kinetics, initially releasing approximately 40% of the tetracycline within the first 24 hours, and then releasing the remaining drug in an almost linear fashion for 7-10 days [12]. In Groups IA and IIA, tetracycline fibers (periodontal AB plus) were mixed with 2 mg of TC gum powder and 0.2 mL of saline and administered as an LDD into the periodontal pockets.

A study was conducted among 40 diabetic patients with periodontitis who either received SRP or SRP with tetracycline fiber as LDD. The clinical parameters evaluated included PI, GI, probing depth, and relative attachment level. C-reactive protein, HbA1c, and lipid profile were the biochemical variables assessed at baseline and one and three months. There was a significant improvement in all clinical parameters, and the HbA1c levels in the tetracycline group asserted its beneficial effects over SRP only [13].

Another study was done among patients with diabetes and moderate-to-severe periodontitis to evaluate if NSPT with adjuvant LDD of tetracycline fibers produces comparable results to surgical therapy. Eighty sites were treated with either tetracycline LDD or flap surgery. The clinical parameters PI, GI, PPD, and clinical attachment loss (CAL) were evaluated at baseline and after four, six, nine, and 12 weeks post-treatment. The study results pointed out NSPT being as effective as surgical therapy pertaining to all the clinical parameters except PPD, for which surgical therapy proved to be superior (p < 0.01) [14].

In this study, both Groups IA and IIA (wherein tetracycline xanthan was administered as an LDD after SRP) showed improvements in GI and PPD from baseline to 12 weeks. Though the PI improved, it was observed only from baseline to eight weeks.

Probiotics are live microorganisms that when ingested confer health benefits to the host. The most commonly used species in probiotic formulations include *Lactobacillus*, *Bifidobacterium*, *Escherichia*, *Enterococcus*, and *Bacillus *genera. The different mechanisms by which probiotics are beneficial to the host include the production of lactic acid and hydrogen peroxide which inhibit the growth and proliferation of bacteria; in addition, they liberate bacteriocins and vitamins which have an anabolic effect on the host [15].

A study evaluated probiotics’ efficacy with professionally administered plaque removal (PAPR) and photodynamic therapy (PDT) in patients with peri-implant mucositis. The test group included PAPR + PDT and professional and home-based administration of probiotics (*Lactobacillus plantarum* and *Lactobacillus brevis*), whereas the control group received PAPR + PDT and administration of a placebo. This study implemented a cross-over design. The clinical variables were assessed before and post-professional treatment protocol after two and six weeks. It was observed that there was no significant difference in treatment outcomes between the groups, negating the role of probiotics as an adjunctive tool [16].

Another study wherein 30 patients with periodontitis participated compared the effects of gel containing aloe vera gel + probiotic (15 patients) versus aloe vera gel alone (15 patients) when used as adjunctive tools after NSPT. It was observed that patients who used aloe vera gel + probiotics achieved superior results, prompting them to be used after NSPT [17].

A meta-analysis aimed to determine the significance of probiotic usage, both as a preventive and therapeutic strategy for periodontitis. Data collected from randomized controlled trials was based on the effects of SRP as a monotherapy in comparison to SRP with adjunctive use of probiotics. CAL and probing depth were assessed as the primary outcomes. Fourteen studies selected showed that adjunctive probiotic therapy after SRP leads to a decrease in probing depth and CAL gain in chronic periodontitis patients. It was observed that probiotics also reduce the bacterial counts of PG and AA when administered for a longer duration [18].

Probiotic + TC which was administered in group IB and group IIB as an adjuvant after SRP showed improvements in GI and PPD from baseline to 12 weeks. The PI, however, showed improvement from baseline to eight weeks only.

On intergroup comparison of the clinical parameters, which were the secondary outcomes, it was observed that PI improved from baseline to eight weeks in all three groups; however, a significant reduction in PI was seen only in Group IIA (controlled diabetics: tetracycline + TC). About GI and PPD, however, although the values improved from baseline to 12 weeks, a significant difference was seen only in Group IIA.

IL1β is observed to participate actively in periodontal tissue destruction and bone resorption. The levels of this cytokine are, therefore, much more frequently detected in the saliva and GCF of patients with periodontitis when compared with healthy individuals [19].

Some studies assessed the influence of SRP on clinical parameters and IL1β, IL8, and MMP-8 levels in GCF in patients with chronic periodontitis. The study population consisted of 30 patients, while 21 periodontally healthy subjects were recruited for the control group. The clinical parameters included PI, GI, PD, and CAL. The levels of IL1β, IL8, and MMP-8 in GCF were measured by ELISA. SRP had a beneficial effect on the clinical and immunological parameters except for CAL four weeks after NSPT in the test group [20].

In this study, IL1β was the primary outcome analyzed. It was observed that the IL1β levels were lowered in the three groups after SRP. However, on intergroup comparison, the values were highly significant only in Group IIA (p < 0.01) (Table 4).

A limitation of the study is that the drug release of the LDDs when mixed with TC gum was not assessed.

## Conclusions

Although all groups showed improvement in clinical and biochemical parameters, superior results were seen in controlled diabetics (Group II), followed by the placebo group (Group III), and insignificant results were seen in the uncontrolled diabetic group (Group I). Thus, the use of LDD systems as adjuncts after SRP may not improve the treatment outcomes in uncontrolled diabetics, which falls in line with the available literature. Among the LDDs, the tetracyclines performed effectively in controlled diabetics when compared to the probiotic group. However, this study paves the way for future studies with a larger sample size that may validate the potential of these treatment strategies effectively.
